# Assessing the effectiveness of garlic (*Allium sativum*) and korarima (*Aframomum corrorima)* against Salmonella spp. and *E. coli* isolates in beef

**DOI:** 10.1038/s41598-026-47468-9

**Published:** 2026-04-21

**Authors:** Degaga Guder Gemeda, Adem Hiko, Bruk Abraha Fitwi, Dereje Regassa Nigussie

**Affiliations:** College of Veterinary Medicine, Haramaya University, P.O. Box 138, Dire Dawa, Ethiopia

**Keywords:** Garlic, Korarima, Air-dried powder, Beef, Bacterial load reduction, In-situ evaluation, Biotechnology, Microbiology

## Abstract

This exploratory in-situ treatment study assessed the effectiveness of garlic (*Allium sativum*) and korarima (*Aframomum corrorima*) against *Salmonella* spp. and *E. coli* isolates from beef in Haramaya town, Eastern Ethiopia. The isolates were prepared at a 7.86 log CFU/g concentration and injected into 100 g of minced meat, followed by inoculation with 1%, 5%, and 10% w/w concentrations of garlic and korarima powders. Both the treatment and control groups were kept at room temperature (22–25 °C) for one, twenty-four, and forty-eight hours. Descriptive statistics such as mean and standard deviation were used to present the data. The garlic-treated *E. coli* decreased by 0.76 log CFU/g for one hour, 1.47 log CFU/g for twenty-four hours, and 1.86 log CFU/g for forty-eight hours. Moreover, garlic decreased *Salmonella* spp. by 0.84 log CFU/g for one hour, 1.04 log CFU/g for twenty-four hours, and 1.11 log CFU/g for forty-eight hours. Korarima powder alone or in combination with garlic showed minimal effects on bacterial load between the treated and untreated groups. The log CFU/g was lower for garlic at 10% and 1% w/w (6.666 ± 0.482 and 7.295 ± 0.163, respectively) than for korarima (7.848 ± 0.044 and 8.006 ± 0.043, respectively) for *E. coli*, while the value for *Salmonella* was (7.662 vs. 8.323) at 1%, (7.346 vs. 8.139) at 5%, and (7.064 vs. 8.201) at 10%. In conclusion, adding garlic to beef can help reduce the amount of *Salmonella* spp. and *E. coli* present in meat when stored naturally at room temperature. Spice-based preservatives can be a good addition to meat microbial safety interventions in low-resource settings.

## Introduction

Foodborne illnesses are still a major public health concern, reinforcing the need for strong food safety measures to protect community health^1^. In the last ten years, there have been severe outbreaks of foodborne illness on every continent, often worsened by increased international trade^2,3^. In developing countries, a combination of limited infrastructure, poor regulatory practices, and dietary habits is responsible for meat-borne illnesses^4,5,6^.

Spices and herbs are used as natural ingredients in the pharmaceutical industry, and their ability to combat pathogens makes them important in the food industry, particularly as food preservatives. Extracts from common spices can inhibit the growth of both pathogenic and spoilage microbes. Thus, preparations considered safe and effective can be used as natural preservatives^7,8,9,10^.

Garlic^11^ and rosemary^12^ extracts were used in the disk diffusion test against *E. coli* and *Staphylococcus aureus*. Using the same method, extracts of basil, thyme, cardamom, cinnamon, mustard, and cloves were also shown to have antimicrobial properties against *Salmonella* Typhi, *S. aureus*,* Pseudomonas aeruginosa*,* Shigella* spp., *Bacillus* spp., and *Streptococcus* spp^13,14^. An in-situ study showed that the antibacterial properties of spices (mustard, coriander, ginger, garlic, basil, and rue) extend the shelf life of *metadata ayib* (Ethiopian cottage cheese)^15^. Though in vitro susceptibility testing is needed in assessing the effectiveness of natural antimicrobial agents^16^, a definitive judgment should be based on evaluation of in vivo (in situ) conditions, which consider the effect of food particles on the antimicrobial activity of test products^17^.

Commercially available essential oils, such as mustard, basil, and oregano, have been shown to have potent antimicrobial properties using qualitative in vitro methods^18,19,7,20,21^. However, when essential oils are combined with food ingredients, their efficacy is reduced^22,23^. Although dried powders are rarely used to assess antimicrobial qualities, a meta-analysis-based literature review found that dried powder inhibits microbial growth just as well as microencapsulated essential oil^24^. As essential oils are expensive to prepare, using dried or freshly made spices is helpful in settings with limited resources.

Ethiopia produces 50 of the 109 spices that are recognized across the globe^25,26^. These spices are widely used for both medical and food preservation purposes^27,28^. Spices are used to produce nearly all popular traditional Ethiopian dishes, mostly for flavor, color, and appetite stimulation. For instance, the spicy sauce made from bird’s eye chili powder, seasoned powdered chili pepper, and mustard seed powder is consumed with uncooked beef roast^29^. The seasoned chili pepper is particularly made from sun-dried pepper and combined with ginger, cardamom, coriander, fenugreek seeds, cloves, cinnamon, garlic, salt, and cayenne pepper. The mixture is then coarsely ground and toasted for a few minutes at low heat, after which it is ground into powder^29,30^.

Despite the use of spices and herbs in meat-based meal preparations, little is known about how these herbs affect microbes in the meat matrix, particularly when stored at room temperature. Research on the in-situ evaluation of spices against meat-borne pathogenic bacteria is crucial to confirm their real-world potential as nature-based solutions to lower the burden of food-related illness. This study was conducted to assess the in-situ effectiveness of air-dried powder of garlic (*A. sativum*) and korarima (*A. corrorima*) against *Salmonella* species and *E. coli* isolates on ground beef at varying durations of room temperature storage conditions.

## Materials and methods

### Study area description

The study was conducted in Haramaya town, which is the administrative center of the Haramaya district, East Hararghe Zone. The town is found approximately 10 km west of the Zonal Administration, Harar, and 516 km east of Addis Ababa (Finfinne), Oromia Regional State. Geographically, this area (Haramaya town) lies between 9°24’0” N latitude and 42°00’1” E longitude at an elevation of 2,047 m above sea level. The town has a municipal abattoir to serve as a slaughterhouse for 16 meat retailers settled in the town, which has a chance of serving (issuing the products) 220,374 people in the district and a minimum of 48,418 people in the town^31^. As a district, there is a population of ruminants used for meat sources: 120,819 cattle, 116,645 goats, and 76,746 sheep. The abattoir located in the town, on average, gets the inputs of the livestock for slaughter per day, about 8 bovine and 8 goat heads for the slaughter services, which can be estimated to be an average of 2,920 heads of bovine and 2,920 heads of goats per year^32^.

### Study design

An exploratory in-situ treatment was conducted in Haramaya University, College of Veterinary Medicine Research Laboratory, from July 2021 to March 2022, to evaluate the in situ antimicrobial property (efficacy) of *Allium sativum* and *Aframomum corrorima* in inhibiting (reducing) the isolated meat-borne zoonotic pathogens. In this exploratory study, there were two bacterial pathogens to be part of the treatment, and beef was bought from butcher shops. According to Humaid et al.^33^., the isolates were subjected to three concentrations (1%, 5%, and 10% w/w) of each of the two basic treatments (garlic and korarima) as well as their mixture in equal amounts (Table 1). The inhibitory effects were evaluated based on ambient temperature storage and different time durations.

**Table 1:** The in-situ treatment design illustration of ground beef treatments with air-dried powder of spices.

Groups	Treatment form	Treatment concentration (wt/wt) per beef		
		Garlic	Korarima	Garlic + Korarima
I	Powder	1 g/100 g	1 g/100 g	1 g/100 g
II	Powder	5 g/100 g	5 g/100 g	5 g/100 g
III	Powder	10 g/100 g	10 g/100 g	10 g/100 g
IV	+ve control	100 g of meat	100 g of meat	100 g of meat
V	-ve control	100 g of sterile meat	100 g of sterile meat	100 g of sterile meat

### Preparation of test organisms

The test organisms were obtained by isolating from butcher shops in the study areas. *Salmonella* was isolated from meat swab samples using the procedure specified by the International Standards Organization (ISO) for *Salmonella* isolation^34^. Moreover, *Escherichia coli* isolation and identification were performed following the guidelines of the International Organization for Standardization^35^.

### Preparation of garlic and korarima powders

#### Garlic preparation

**Fig. 1:**
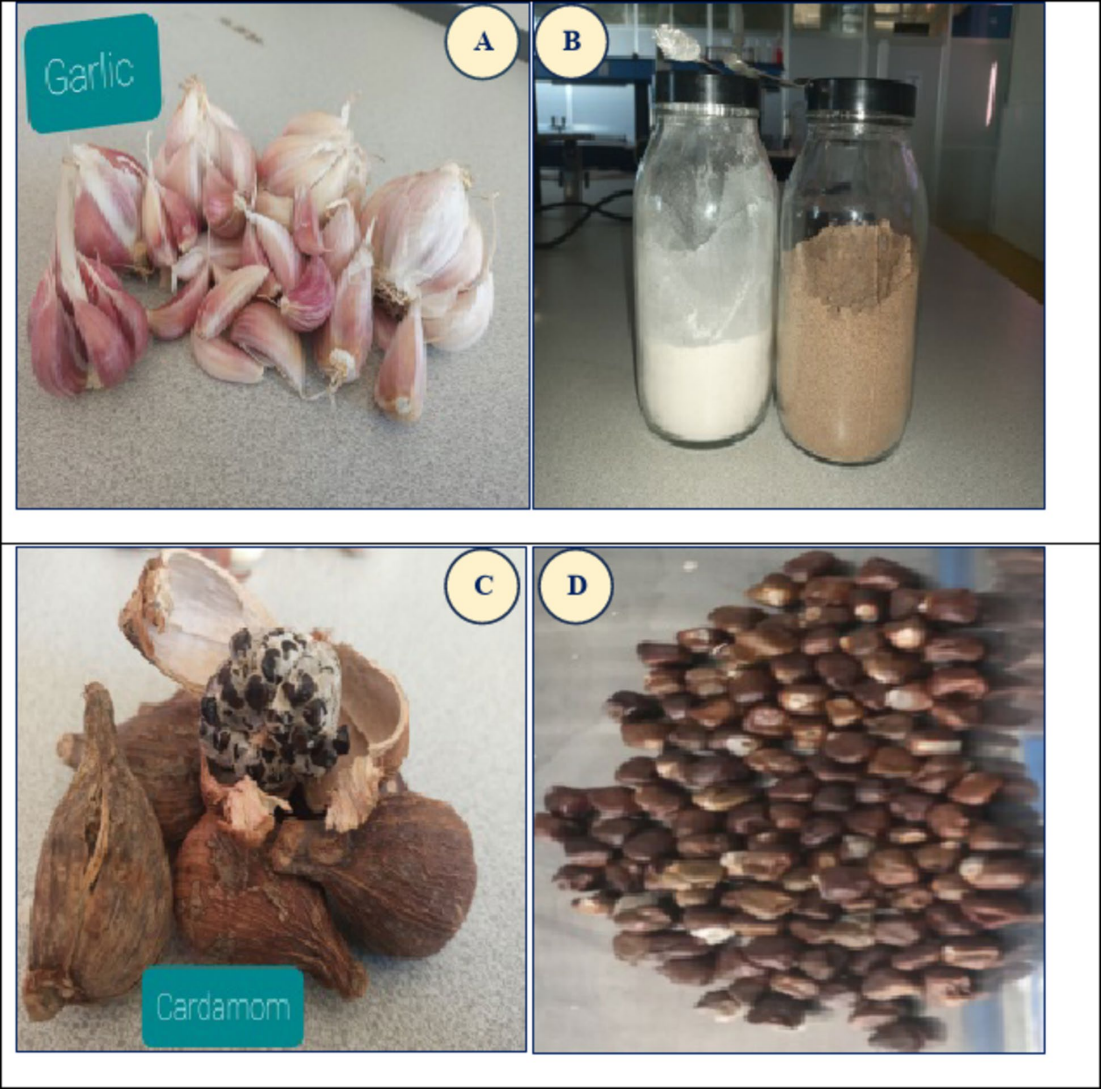
Preparation of spices: **A** (garlic cloves); B (Garlic and korarima powder left to right); C (Korarima bulb); D (Korarima seeds).

The protocol of Rahman et al.^36^ was followed to prepare the garlic powder. Figure 1 shows the preparation of spices. Fresh garlic (*Allium sativum*) was bought from a local market (Harar, Dakar) and stored at room temperature until used for the experiments, and bulbs of garlic with multiple cloves (Fig. 1A) were prepared for peeling without damage. About five (5) bulbs were weighed (59.27 g, 55.96 g, 41.20 g, 30.64 g, and 20.74 g), making up 20, 22, 11, 14, and 12 cloves, respectively (Table 2). Each of the bulbs was separated into individual garlic cloves, and the cloves were peeled, and the one with a visible abnormality (damaged) was discarded. To prepare garlic powder, each clove is sliced into thin strips along the longitudinal axis and spread on a stainless-steel mesh tray. The shaded dried slices were ground with an electrical kitchen grinder to form a powder and stored in sealed containers (Fig. 1B) by providing information and preventing moisture absorption and contamination from mold at 4 °C until used for the desired experimental activity.

**Table 2:** General Information Regarding the Weight of Used Garlic.

Bulb (in g)	No of cloves	Random weight of cloves in g
59.27	20	6.34, 2.52, 1.4
55.96	22	3.7, 1.9, 1.74
41.20	11	6.5, 2.01, 1.2
30.64	14	3.79, 1.48, 1.4
20.74	12	2.37, 1.28, 1.04

#### Korarima preparation

To prepare the korarima powder, the same protocol was followed as in the preparation of garlic. The plant seeds (fruit) of Korarima (*Aframomum corrorima*) were also obtained from the local market (same market as garlic), and the average mass of the bulb was measured as 9.11 g, 6.36 g, and 5.67 g and then peeled off and dried by the shaded air-dry method, and the bulbs of Korarima containing the seeds were prepared for the collection of the seeds in their natural state (Fig. 1C). The seeds of Korarima were cleaned of unnecessary materials from the bulb contents and prepared for grinding (Fig. 1D). They were ground by an electrical grinder to obtain Korarima’s powder, and the powder was then stored (Fig. 1B) as described for garlic in the refrigerator at 4 °C for the experimental work.

### Antibacterial tests

#### Meat samples treatment with herbs/spices

The current study protocol was adopted from the method described by Humaid et al.^33^. The beef obtained (purchased) from retail shops was rubbed using alcohol (70% ethyl alcohol) to avoid surface contamination. After proper evaporation of the rubbed alcohol, the external part of the meat was trimmed off with a sterile knife, while the rest was prepared for treatment. The beef was contaminated with *E. coli* and *Salmonella*. Thus, three-level concentration treatments (1, 5, and 10% w/w) of each and an equal proportion of the mixture were applied aseptically on 100 g of the meat. The meat was arranged into the following treatment groups:

Treatment 1 (T1): 1 gram, 5 g, and 10 g of each, and a mixture of spices added individually to *100 g* of sterile beef that was contaminated separately by *E. coli* and *Salmonella* spp., with adjusted half turbidity by comparing with the standard McFarland solution.

Treatment 2 (positive control): *100 g* of sterile beef contaminated with half the adjusted turbidity of each bacterial pathogen mentioned above for growth control.

Treatment 3 (negative control): *100 g* of sterile meat (beef) without the addition of any treatments or pathogens. This stands for a control treatment (negative control), which was used to control the sterility of the test.

Each treatment (which is described under T1) was then incubated at room temperature (22–25 °C) for the desired period, transferred into a sterile stomacher bag, and evaluated at three (3) time intervals: 1 h, 24 h, and 48 h.

#### Total viable count

All treatments were subjected to viable cell count as described earlier by Hattab et al.^37^. After incubation for the specified period, sterile distilled water was added to the meat treatment in a stomacher’s bag, and the mixture was then homogenized in a stomacher (240 rpm per minute) for 2 min. All the prepared treatments with targeted pathogens were subjected to serial dilutions, followed by the immediate spread of 0.1 ml (100 µl) of these dilutions by using a sterile glass spreader on the surface of pre-dried Eosin Methylene Blue (EMB) agar and Xylose Lysine Deoxycholate (XLD) agar medium, incubated at 37 °C for 24 h for viable counts of *E. coli* and *Salmonella* spp., respectively. The colonies with a metallic sheen appearance on EMB and red color with a black center on XLD were considered to count *E. coli* and *Salmonella*, respectively. Colony-forming unit per milliliter of meat suspension (CFU/g) was calculated as the average number of colonies from triplicated plates divided by the dilution factor, then multiplied by volume plated.

### Data management and analysis

The collected data were handled and entered in a Microsoft Excel spreadsheet and analyzed using Statistical Packages for Social Sciences (SPSS) version 20 statistical software. Since we used only technical replications, results were expressed as mean ± standard deviation (M ± SD).

## Results

### Effects of garlic and korarima on e. coli load reduction

**Fig. 2:**
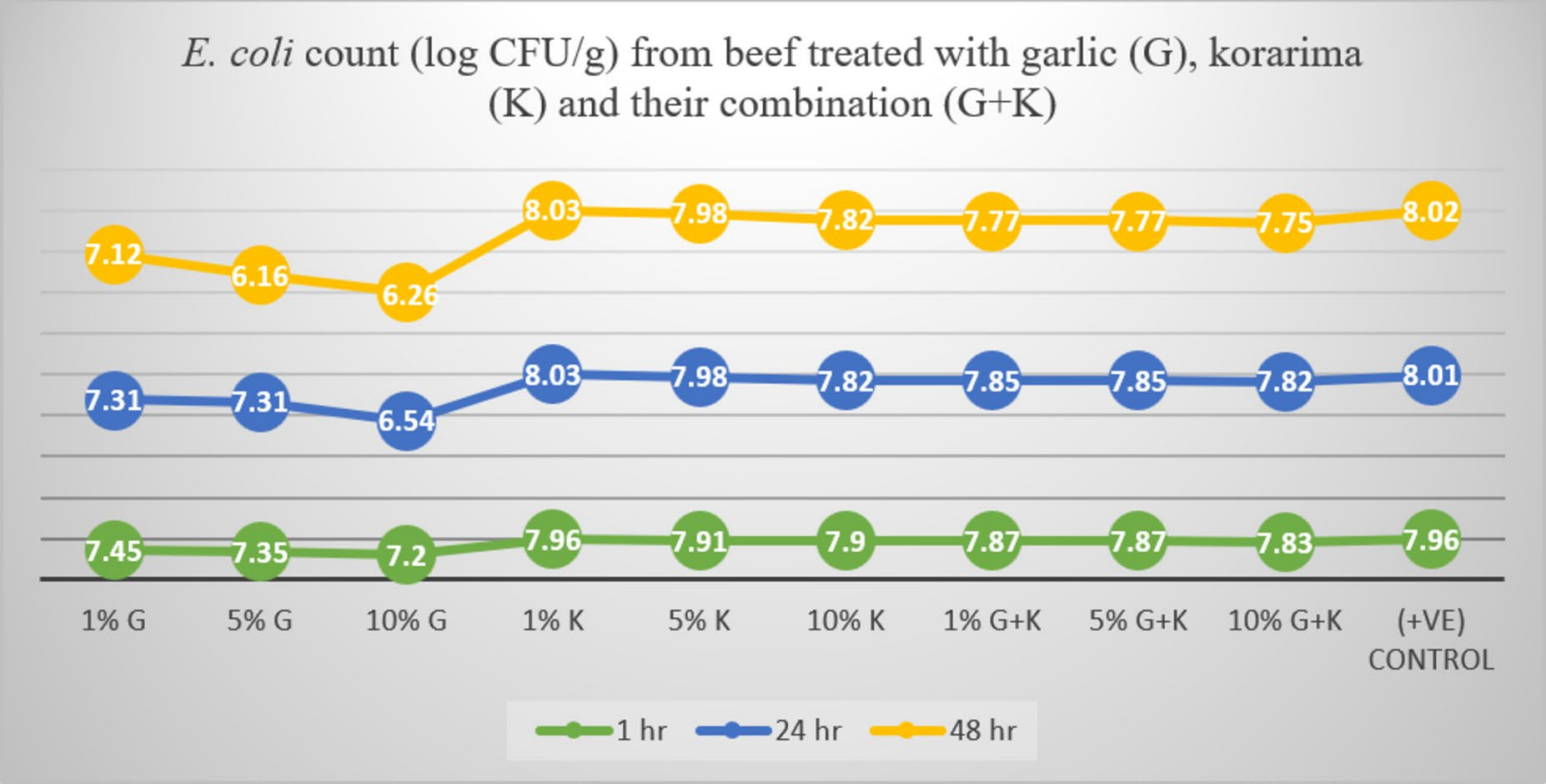
Log CFU/g *E. coli* count from beef treated with different preparations and concentrations of air-dried garlic and korarima powder.

The mean log CFU/g *E. coli* load as a function of different concentrations of the used spices at different durations of treatment is presented in Fig. 2**and** Table 3. Garlic treatment at 5% achieved the best *E. coli* log reduction (6.16 ± 0.275), followed by 10% concentration at treatment durations of 48 h (6.26 ± 0.241) and 24 h (6.54 ± 0.278). Another notable log reduction (7.12 ± 1.74) was recorded for garlic at 1% concentration after 48 h of exposure (Table 3).

**Table 3:** Total *E. coli* count (Mean ± SD) from treated beef with different treatment types and different concentration levels.

Treatments	Concentrations (w/w)	Log CFU/g at:		
		1 h	24 h	48 h
Garlic	1%	7.45 ± 0.178	7.31 ± 0.243	7.12 ± 174
	5%	7.35 ± 0.289	7.31 ± 0.185	6.16 ± 0.275
	10%	7.20 ± 0.133	6.54 ± 0.278	6.26 ± 0.241
Korarima	1%	7.96 ± 0.008	8.03 ± 0.044	8.03 ± 0.021
	5%	7.91 ± 0.011	7.98 ± 0.130	7.98 ± 0.067
	10%	7.90 ± 0 0.014	7.82 ± 0.256	7.82 ± 0.223
Garlic + Korarima	1%	7.87 ± 0 0.066	7.85 ± 0.097	7.77 ± 0.232
	5%	7.87 ± 0 0.066	7.85 ± 0.122	7.77 ± 0.230
	10%	7.83 ± 0.134	7.82 ± 0.152	7.75 ± 0.255
Positive control		7.96 ± 0.027	8.01 ± 0.052	8.02 ± 0.034

The effect of garlic treatment on *E. coli* was compared with other treatments at the same concatenation levels (Table 4**)**. The *E. coli* reduction effect of garlic was better than any treatment based on korarima.

**Table 4:** Difference in mean log CFU/g *E. coli* load at different treatment types of the same concentrations.

Spices used		Mean log count	N	SD	Pair	*E. coli* log difference (± SD)
Pair 1	1%G	7.295	3	0.163	1%G − 1%K	-0.711(0.21)
	1%K	8.006	3	0.043		
Pair 2	5%G	6.887	3	0.638	5%G − 5%K	-1.071(0.67)
	5%K	7.957	3	0.043		
Pair 3	10%G	6.666	3	0.482	10%G − 10%K	-1.182(0.44)
	10%K	7.848	3	0.044		
Pair 4	1%G	7.295	3	0.163	1%G − 1%G + K	-0.534(0.11)
	1%G + K	7.829	3	0.053		
Pair 5	5%G	6.887	3	0.638	5%G − 5%G + K	-0.941(0.59)
	5%G + K	7.828	3	0.05		
Pair 6	10%G	6.666	3	0.482	10%G-10%G + K	-1.133(0.45)
	10%G + K	7.799	3	0.040		

N = Number of triplicate counts; SD = Standard deviation; G=Garlic, K= Korarima, G + K = Garlic and Korarima.

### Effects of garlic and korarima on salmonella load reduction

**Fig. 3:**
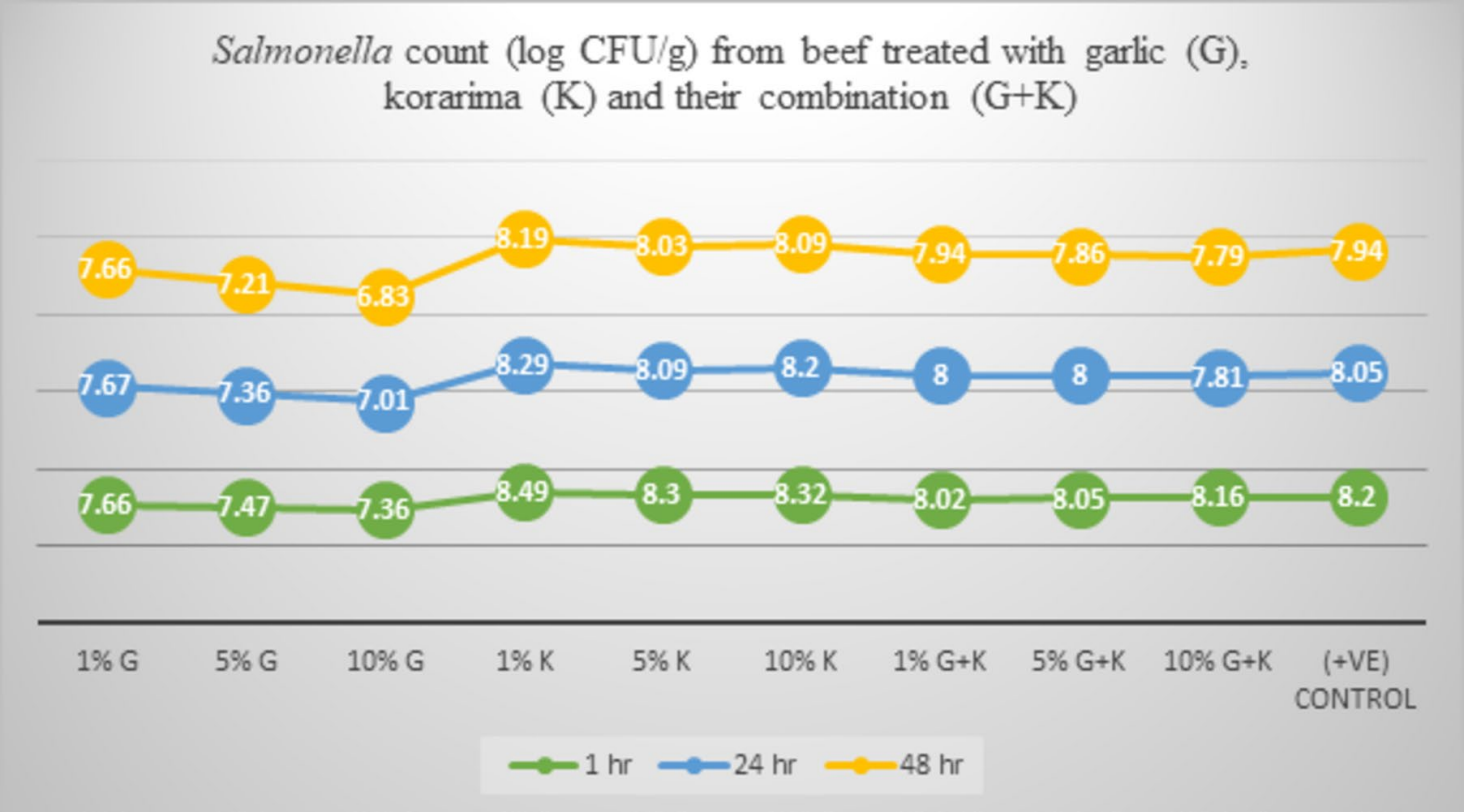
Log CFU/g *Salmonella* count from beef treated with different preparations and concentrations of air-dried garlic and korarima powder.

The mean log CFU/g *Salmonella* load as of different concentrations of the used spices at different durations of treatment is presented in Fig. 3**and** Table 5. Garlic treatment at 10% concentration achieved the best *Salmonella* reduction at all treatment durations, such as 7.36 ± 0.141 (1 h), 7.01 ± 0.291 (24 h), and 6.83 ± 0.718 (48 h).

**Table 5:** Total *Salmonella spp.* count (mean ± SD) from treated beef with different treatment types and different times of exposure.

Treatment	Concentration (w/w)	Log CFU/g at:		
		1 h	24 h	48 h
Garlic	1%	7.66 ± 0.158	7.67 ± 0.066	7.66 ± 0.037
	5%	7.47 ± 0.187	7.36 ± 0.119	7.21 ± 0.147
	10%	7.36 ± 0.141	7.01 ± 0.291	6.83 ± 0.718
Korarima	1%	8.49 ± 0.023	8.29 ± 0.156	8.19 ± 0.416
	5%	8.30 ± 0.084	8.09 ± 0.145	8.03 ± 0.036
	10%	8.32 ± 0.132	8.20 ± 0.122	8.09 ± 0.317
Garlic + Korarima	1%	8.02 ± 0.161	8.00 ± 0.011	7.94 ± 0.329
	5%	8.05 ± 0.213	8.00 ± 0.083	7.86 ± 0.089
	10%	8.16 ± 0.161	7.81 ± 0.041	7.79 ± 0.081
Control (+)		8.20 ± 0.139	8.05 ± 0.169	7.94 ± 0.278

Regardless of treatment duration, the effect of garlic treatment on *Salmonella* was superior to that of korarima or their combination at the same concentration (Table 6**)**. In addition, a substantial difference (> 1-log) was seen at 10% concentration, while notable variations (> 0.5-log) occurred at most other levels. Negligible variation was only seen at 1% concentration of garlic and its combination (Table 6). Regardless of treatment concentration, there was no observed notable difference (< 0.5-log) across all paired treatment durations (Table 7).

**Table 6:** Difference in mean log CFU/g *Salmonella* load at different treatment types of the same concentrations.

Spices used		Mean log count	N	SD	Pair	*Salmonella* log difference (± SD)
Pair 1	1%G	7.662	3	0.006	1%G-1%K	-0.661 (0.149)
	1%K	8.323	3	0.153		
Pair 2	1%G	7.662	3	0.006	1%-1%G + K	-0.328 (0.036)
	1%G + K	7.989	3	0.041		
Pair 3	5%G	7.346	3	0.133	5%G-5%K	-0.793 (0.055)
	5%K	8.139	3	0.141		
Pair 4	5%G	7.346	3	0.133	5%G-5%G + K	-0.624 (0.037)
	5%G + K	7.970	3	0.101		
Pair 5	10%G	7.064	3	0.269	10%G-10%K	-1.137 (0.159)
	10%K	8.201	3	0.114		
Pair 6	10%G	7.064	3	0.269	10%G-10%G + K	-0.855 (0.092)
	10%G + K	7.919	3	0.209		

N = Number of triplicate counts; SD = Standard deviation; G=Garlic; K = Korarima; G + K = Garlic and Korarima.

**Table 7:** Difference in mean log CFU/g *Salmonella* load at various holding times of spice treatments.

Duration of treatment		Mean log count	N	SD	Pair	*Salmonella* log difference (± SD)
Pair 1	1hrG	7.497	3	0.155	1hrG − 24hrG	0.153(0.181)
	24hrG	7.345	3	0.330		
Pair 2	1hrG	7.498	3	0.155	1hrG − 48hrG	0.268(0.260)
	48hrG	7.229	3	0.414		
Pair 3	24hrG	7.345	3	0.330	24hrG − 48hrG	0.115(0.091)
	48hrG	7.229	3	0.414		
Pair 4	1hrK	8.367	3	0.105	1hrK − 24hrK	0.173(0.048)
	24hrK	8.195	3	0.100		
Pair 5	1hrK	8.367	3	0.105	1hrK − 48hK	0.266(0.037)
	48hK	8.101	3	0.080		
Pair 6	24hrK	8.195	3	0.100	24hrK − 48hK	0.094(0.026)
	48hK	8.101	3	0.080		
Pair 7	1hrG + K	8.078	3	0.073	1hrG + K -24hrG + K	0.141(0.185)
	24hrG + K	7.938	3	0.113		
Pair 8	1hrG + K	8.078	3	0.073	1hrG + K -48hrG + K	0.215(0.147)
	48hrG + K	7.863	3	0.076		
Pair 9	24hrG + K	7.938	3	0.113	24hrG + K -48hrG + K	0.074(0.065)
	48hrG + K	7.863	3	0.077		

N = Number of triplicate counts; SD = Standard deviation; G=Garlic; K= Korarima; G + K= Garlic combined with Korarima.

### Load reduction differences between e. coli and salmonel*la*

The difference in log reduction between *E. coli* and *Salmonella* was most notable at 1% and 5% garlic (~ 0.7 log), while at other treatments, the variation was negligible (Table 8).

**Table 8:** Differences in mean log CFU/g *E. coli* Versus *Salmonella* load of similar concentration of spice treatments.

Paired groups		Mean log count	N	SD	Difference	Log difference (± SD)
Pair 1	1%GE	7.00	3	0.000	1%GE − 1%GS	− 0.662 (0.006)
	1%GS	7.66	3	0.006		
Pair 2	5%GE	6.67	3	0.577	5%GE − 5%GS	− 0.679 (0.459)
	5%GS	7.35	3	0.133		
Pair 3	10%GE	6.67	3	0.577	10%GE − 10%GS	− 0.397 (0.412)
	10%GS	7.06	3	0.2693		
Pair 4	1%KE	8.00	3	0.000	1%KE − 1%KS	− 0.323 (0.153)
	1%KS	8.32	3	0.153		
Pair 5	5%KE	8.00	3	0.000	5%KE − 5%KS	− 0.139 (0.141)
	5%KS	8.14	3	0.141		
Pair 6	10%KE	8.00	3	0.000	10%KE − 10%KS	− 0.201 (0.114)
	10%KS	8.20	3	0.114		

*N = Number of triplicate counts; SD = Standard deviation; GE=Garlic on *E. coli*; GS= Garlic on *Salmonella*; KE= Korarima on *E. coli;* KS= Korarima on *Salmonella*.

## Discussion

Even though the concentration of active ingredients in essential oils and spice extracts contributes to the potency against bacterial agents^38,39,40^, the dried powder inhibits microbial growth just as well as essential oils^24^. Using varying concentrations and storage times (up to two days) at room temperature (22–25 °C), the current study showed the effects of air-dried garlic powder, korarima, and proportionate mixtures of the two on the reduction of *E. coli* and *Salmonella* on ground beef.

In our study, garlic powder showed a dose-dependent antimicrobial effect against *E. coli* in contaminated beef stored at room temperature. After 1 h, garlic at 1%, 5%, and 10% w/w reduced *E. coli* by 45.9%, 54.9%, and 68.4% (to log CFU/g 7.45, 7.35, and 7.20, respectively, vs. 7.96 in control). At 24 h, 1% and 5% both reduced counts by 63% (7.31 log), while 10% achieved a 72.8% reduction (6.54 log vs. 8.01 control). By 48 h, reductions reached 81% (7.12 log) at 1%, 92% (6.16 log) at 5%, and 87.1% (6.26 log) at 10%, compared to 8.02 log in controls. Agreeably, Wawrzyniak and Drozdzynska^41^ indicated that it was the extract concentration that significantly influenced *E. coli* growth, rather than the drying temperature of garlic.

In line with these findings, earlier studies on oven-dried garlic powder achieved 1–2.8 log₁₀ CFU/g reductions in *E. coli* under cold storage for 6 days, while oven-dried garlic powder achieved 0.6–1.0 log₁₀ CFU/g significant reductions after two days^23^. Fresh garlic paste showed strong antimicrobial activity (about 1 log CFU/g reduction) on *E. coli* O157:H7 at 3 days at 4 °C and 8 °C on ground beef^42^. Likewise, fresh garlic showed a significant reduction, such as > 3 log₁₀ CFU/g, under refrigeration at 4 °C^43^. The efficacy of garlic against *E. coli* on other food products, such as fermented cottage cheese, was reported in Ethiopia^15^. While various preparations are affecting allicin^23^, freeze-drying has better retention of this active ingredient than drying^44,45^. The current findings agree with the study conducted by Hattab et al.^37^, which showed the potential of garlic powder in reducing the load of *E. coli* O125 from contaminated meat, in which the efficacy of the treatment increased as the days of preservation prolonged, and the concentration of garlic powder. Moreover, reduction intensification with extended storage has been previously reported, but with the lower reduction percentage being recorded at increased concentrations (3% vs. lower concentrations) of garlic powder applied on chicken meat with 96 h of h storage^37^. The current greatest reduction at 5% (92%) surpasses that of 10% at 48 h, suggesting diminishing antimicrobial returns beyond a certain threshold, which is not in line with earlier findings^43,46^. Thus, the plateau at higher doses could reflect random error rather than microbial adaptation or garlic’s own decomposition, as it was reported that it can be stored for months at ambient temperature for months^47^.

In the current study, doubling the garlic concentration (5% to 10%) did not result in substantial variability in reducing *E. coli* (< 1.0-log). This suggests that the bioactive compounds in garlic powder are highly potent even at lower concentrations. In their study, Octavia et al.^48^ reported no significant differences (*p* > 0.05) between the viability values for 25%, 50%, and 100% garlic extracts on *E. faecalis*. Also, allicin’s most effective nature against *E. coli* has been shown by an observed lowest MIC value (0.125 µg/mL) compared to other organisms^49^.

The mean log CFU/g *E. coli* load difference between garlic and korarima-treated beef at similar concentrations showed an overall better reduction effect of garlic than korarima. Except at 5% garlic vs. 5% korarima (with a mean difference of -1.071 ± 0.67), other concentration combinations showed a better *E. coli* reduction effect of garlic than korarima. Combined effects of spices (garlic + korarima) on *E. coli* showed log counts of 7.799 ± 0.040 log CFU/g at 10% and 7.828 ± 0.05 log CFU/g at 1 and 5% concentrations, which were like the positive control. This finding shows that korarima is not effective against the test organisms, which is in line with the observation of Adedayo et al.^50^, in which methanolic extracts of *A. melegueta* (the same family as *A. corrorima*) showed no significant effects on *E. coli* and other gram-positive bacteria. In contrast, Bacha et al.^39^ from Ethiopia reported the antibacterial (*E. coli* and *P. aeruginosa*) and antifungal (*C. albicans*) activity of korarima essential oils. With research primarily noting antioxidants rather than potent antibacterial effects^51,52^, little attention is given to the antimicrobial effect of korarima on meat preparations.

In the current study, garlic remarkably (> 1.0 log) reduced *Salmonella* in minced meat at 5% and 10% w/w after 24 and 48 h. Likewise, the effects of garlic on *Salmonella* with a subsequent increase in its concentrations were notable (> 0.5-log reduction). These findings agree with the reports of Humaid et al.^33^ and Rahman et al.^36^. Increased concentrations of fresh garlic (3–5% vs. 2%) and garlic oil (120–200 mg/kg vs. 80 mg/kg) achieved > 3 log CFU/g reductions in *Salmonella* Typhimurium, *E. coli*, and *L. monocytogenes* in ground mutton after an extended duration of storage at 4 °C^53^. On the other hand, unlike the findings on *E. coli*, it was seen that the maximum duration of exposure (i.e., 48 h) did not achieve a better reduction in *Salmonella*. A similar trend was reported by Uhart et al.^54^, in that garlic paste at 5% w/w concentration achieved a better reduction of S. *Typhimurium* in ground beef during 3 days of storage than shorter and extended durations (such as 7–10 days). Generally, *E. coli* is more sensitive to garlic than *Salmonella*^36,33^. A meta-analysis-based report shows that garlic significantly reduces coliforms, *Staphylococcus*, *E. coli*, *L. monocytogenes*, and fungi, but with a lowered effect on *Salmonella*, which suggests that the lower number of studies on *Salmonella* might affect the concluding remarks^24^.

The current study has several limitations. These include a lack of independent replications that consider new batches of beef samples and bacterial isolates, experimenting with different temperature ranges, and considering beef matrices with different pH and other relevant physicochemical parameters.

## Conclusion

Based on this in situ evaluation, *A. sativum* (garlic) powder applied at 10% w/w of beef substantially reduces (> 1 log) *E. coli* and *Salmonella* counts when incubated for 24–48 h at ambient temperature (22–25 °C). *A. corrorima* (korarima) powder, whether used alone or combined with garlic, showed no notable inhibition (< 0.5-log reduction) compared to the positive control. A substantial difference (> 1-log) in *E. coli* and *Salmonella* reduction was seen between garlic and korarima at the 10% level. Also, a remarkable difference in *E. coli* reduction (~ 1.1 log) was noted at 5% preparations of the powders. This finding implies that garlic powder may have bioactive chemical compounds with therapeutic potential to extend the shelf life of foods when evaluated at room temperature storage for an extended period. The findings of this study provided evidence to encourage the standardized use of spices for the development of novel antibacterial formulations with stronger efficacy that can be used as alternatives for food storage and guarantee food safety by inhibiting zoonotic bacteria growth. Future investigations should explore garlic’s combined effects with refrigeration to refine its use as a natural preservative in meat supply chains.

## Data Availability

The data of this study are available from the corresponding author upon request. We confirm that the copy of this manuscript is uploaded to the online library (Institutional website) of Haramaya University and can be retrieved by this address ( **Date: ** 2022, **URI: ** http://ir.haramaya.edu.et//hru/handle/123456789/5044 ) .
